# Graduates in Medicine in the United States

**Published:** 1888-04

**Authors:** 


### *GRADUATES IN MEDICINE IN THE UNITED STATES.*

Dr. Perry H. Millard, of Minnesota (*Journal American Medical Association*) says: We have in the practice of medicine in the United States to-day one physician to every 755 inhabitants. They have in England and Hungary one physician to every 1,666 inhabitants; in Italy, one physician to every 1,639 inhabitants; in Austria, one physician to every 2,932 inhabitants; in Germany, one physician to every 3,225 inhabitants; in France, one physician to every 3,780 inhabitants; in Sweden, one physician to every 7,909 inhabitants. It will, therefore, be seen that the proportion of physicians to the population is ten times as great in this country as in Sweden, four times as great as in France, Germany and Austria, and twice as great as in Great Britain, Italy and Hungary.

The number of medical schools in this country is on the increase, and this increase is not in response to the demands of either the profession or public. Of the 130 medical institutions in this country, less than one dozen are in any way endowed. Seventy-nine colleges of this country graduate men upon two courses of lectures and three years of pupilage.

